# IL-12 as a Potential Prognostic Marker in Penile Cancer: Implications for Immune Dysregulation

**DOI:** 10.3390/ijms262411829

**Published:** 2025-12-07

**Authors:** Mateusz Czajkowski, Michał Kunc, Jacek Kieżun, Bartłomiej Emil Kraziński, Marcin Matuszewski, Weronika Łyzińska, Oliver W. Hakenberg, Piotr M. Wierzbicki

**Affiliations:** 1 Department of Urology, Medical University of Gdańsk, Mariana Smoluchowskiego 17 Street, 80-214 Gdańsk, Poland; 2 Department of Pathomorphology, Medical University of Gdańsk, Mariana Smoluchowskiego 17 Street, 80-214 Gdańsk, Poland; 3 Department of Anatomy and Histology, School of Medicine, Collegium Medicum, University of Warmia and Mazury in Olsztyn, Warszawska 30, 10-082 Olsztyn, Poland; 4 ED Scientific Circle of Pathomorphology, Medical University of Gdańsk, Dębinki, 80-211 Gdańsk, Poland; 5 Department of Urology, Jena University Hospital, 07747 Jena, Germany; 6 Department of Histology, Medical University of Gdańsk, Dębinki, 80-211 Gdańsk, Poland

**Keywords:** penile cancer, squamous cell carcinoma, cytokines, IL-12, prognosis, biomarker

## Abstract

Penile cancer (PeCa) is a rare malignancy with few validated tissue biomarkers to guide prognosis and treatment, despite growing evidence for a key role of inflammation in its biology. This retrospective study evaluated whether the immuno-expression of selected pro-inflammatory cytokines is associated with disease progression and cancer-specific survival (CSS) in PeCa. Immunohistochemistry (IHC) analysis of eight cytokines (IL-1A, IL-1B, IL-2, IL-6, IL-12, TGF-β1, TNF-α and IFN-γ) was performed in paired tumour tissues and corresponding negative surgical margins from 94 patients with penile squamous cell carcinoma. Compared with surgical margins, tumour tissues showed a characteristic inflammatory shift, with markedly increased IL-1β and IL-6 and relatively reduced TNF-α, IFN-γ, IL-12 and IL-2. Receiver Operating Characteristic (ROC) analysis indicated that TNF-α, IL-6 and IL-12 had the strongest ability to discriminate tumour from normal tissue and provided data-driven cut-offs for subsequent analyses. Within tumour samples, high IL-1α, IL-12 and TGF-β1 immuno-expression was significantly associated with advanced UICC TNM prognostic stage and lymph node involvement. Importantly, in contrast to the classically anti-tumour role of IL-12 described in many other solid cancers, increased IL-12 immuno-expression in tumour tissues in our cohort was independently associated with poorer CSS in multivariable Cox regression (HR 2.42, 95% CI: 1.08–5.41, *p* = 0.031), alongside advanced TNM stage (HR 5.03, 95% CI: 2.12–11.95, *p* = 0.0002). These findings highlight IL-1α, IL-12 and TGF-β1 as promising tissue biomarkers of aggressive PeCa and support a central role for cytokine-driven immune dysregulation in penile cancer. The prognostic value of IL-12 should be considered exploratory and warrants validation in larger, multicentre cohorts.

## 1. Introduction

Penile cancer (PeCa) is a rare malignancy in Europe and the United States, with an incidence ranging from 0.1 to 1 case per 100,000 males. However, it exhibits a higher prevalence in certain countries in Africa, Asia, and South America, where it constitutes up to 10% of all cancer cases [1,2]. Despite its low incidence in developed nations, the global impact of PeCa remains substantial. In 2020, the worldwide incidence and mortality rates of PeCa were 36,068 and 13,211, respectively [3]. Squamous cell carcinoma (SCC) is the most prevalent histopathological type, with 14 different subtypes, accounting for 95% of all PeCa cases. Research has identified several etiological factors, including phimosis, chronic inflammation of the glans and foreskin (associated with phimosis, such as lichen sclerosus), human papillomavirus (HPV) infection, and tobacco use [2,4].

The prognosis of PeCa predominantly depends on the stage at the time of diagnosis. Patients diagnosed with PeCa in stages I and II demonstrate a significantly favourable prognosis, with a five-year survival rate of up to 85% following surgical treatment. In contrast, in stages III and IV, the survival rate decreases to 59% and 11%, respectively [5]. Consequently, early detection is crucial for improving patient outcomes. However, only a few widely recognised biomarkers have been reported that may facilitate early diagnosis and influence the prognosis of patients with PeCa.

Inflammation has been recognised as a factor in the development and progression of various cancers, particularly those affecting the urogenital system [6]. There is a well-documented association between chronic inflammation and bladder cancer development, with causative factors such as urogenital schistosomiasis or long-term indwelling catheters. The mechanisms through which schistosomiasis-related inflammation induces bladder carcinogenesis are not fully understood. Chronic inflammation results in the generation of reactive oxygen and nitrogen species, which can induce DNA damage and genomic instability [7]. Moreover, the inflammatory environment stimulates cell proliferation, angiogenesis, and the accumulation of genetic alterations. Inflammatory factors also substantially influence the development of renal cell carcinoma (RCC). Recent studies have highlighted the prognostic significance of pro-inflammatory molecules in RCC. Elevated levels of specific inflammatory markers, such as interleukin 6 (IL-6) and tumour necrosis factor-alpha (TNF-α), have been consistently associated with poor clinical outcomes [8,9].

According to a recent review [10], inflammatory processes play a significant role in the initiation, progression, and metastasis of PeCa. The PeCa tumour microenvironment, characterised by a high concentration of inflammatory cells and pro-inflammatory cytokines, facilitates the accumulation of epithelial mutations. It also promotes angiogenesis, cellular migration, and metastasis [10]. Moreover, a recent study demonstrated the elevated expression of pro-inflammatory cytokines in PeCa, including interleukins 1A (IL-1A) and 1B (IL-1B), IL-6, interferon γ (IFN-γ), and transforming growth factor β (TGF-β). While no overall association was found between cytokine progression and patient survival, a positive correlation was observed specifically between IFN-γ expression and disease progression. However, the preliminary nature of the study and its limited sample size (6 PeCa patients and 13 controls) require further investigation to validate these findings [11]. Despite the increasingly recognised role of inflammation in PeCa, the specific contributions of pro-inflammatory cytokines to disease progression and prognosis remain unclear. This limitation is particularly significant given the existing data showing increased levels of pro-inflammatory cytokines—such as IL-1A, IL-6, and IFN-γ—in patients with lichen sclerosus and phimosis, both of which are well-established risk factors for PeCa [12]. Therefore, elucidating the molecular mechanisms linking inflammation to PeCa is essential for a better understanding of PeCa biology, developing targeted prevention strategies, identifying novel biomarkers, and introducing innovative therapeutic approaches.

The aim of this study was to assess the immuno-expression of IL-1A, IL-1B, IL-2, IL-6, IL-12, TGF-β1, TNF-α, and IFN-γ in PeCa tissues and the corresponding negative surgical margins. Furthermore, the study investigated the correlation between cytokine expression levels and both disease progression and cancer-specific survival (CSS). By evaluating the expression of these cytokines, the study sought to identify potential biomarkers for early diagnosis and novel targets for therapeutic intervention in PeCa. To achieve this objective, immunohistochemistry (IHC) analysis was performed on tissue samples from 94 patients diagnosed with penile cancer. The results demonstrated that elevated expression of IL-1α, IL-12, and TGF-β1 in tumour tissues was significantly associated with advanced stage and lymph node involvement. Importantly, high IL-12 immuno-expression emerged as an independent predictor of poor CSS, suggesting that IL-12 may serve as a valuable prognostic biomarker and a potential therapeutic target in the context of penile cancer.

## 2. Results

### 2.1. Patient Characteristics and Oncological Outcomes

The study included 94 male patients, all of whom were of White Caucasian ethnicity, with a median age of 64 years (range: 34–84) and a median body mass index (BMI) of 29 kg/m^2^ (range: 17–38). The median follow-up duration was 36.5 months (range: 3–138 months). At the time of analysis, 59 patients (62.8%) were alive, while 35 (37.2%) had died. PeCa was the predominant cause of mortality, accounting for 30/35 (85.71%) cases. Other causes of death included heart failure (*n* = 2, 5.71%), stroke (*n* = 1, 2.86%), COVID-19 (*n* = 1, 2.86%), and colon cancer (*n* = 1, 2.86%).

Histopathological examination, performed according to the 2022 WHO Classification of Tumours of the Urinary System and Male Genital Organs [13], confirmed the presence of the usual type of SCC in all patients. The specimens were categorized as penile intraepithelial neoplasia (PeIN) grade III (*n* = 7, 7.45%), well-differentiated G1 (*n* = 21, 22.34%), moderately differentiated G2 (*n* = 49, 52.12%), and poorly differentiated SCC G3 (*n* = 17, 18.09%). Furthermore, p16 status which serves as surrogate marker for transcriptionally active high-risk HPV, was positive in 10 patients (10.64%) and negative in 84 patients (89.36%).

Pathological staging (TNM) was PeIN in 7 cases (7.45%), pT1a in 29 (30.85%), pT1b in 13 (13.83%), pT2 in 32 (34.04%), pT3 in 12 (12.77%), and pT4 in 1 case (1.06%). Regarding lymph node involvement, the pN stages were as follows: pNx in 10 patients (10.63%), pN0 in 50 (53.2%), pN1 in 10 (10.63%), pN2 in 12 (12.77%), and pN3 in 12 patients (12.77%). Distant metastasis was identified in one patient (1.06%) (see Table 1).

### 2.2. Evaluation of Immunohistochemistry Reactions in All Samples

IHC staining was performed for eight cytokine proteins—TNF-α, IFN-γ, TGF-β1, IL-1α, IL-1β, IL-2, IL-6, and IL-12—in paired PeCa tumour samples and corresponding negative-margin tissue. Consistent results were achieved for all 94 paired tumour–normal samples for TNF-α and IFN-γ, for 93 paired samples for TGF-β1 and IL-1α, for 90 paired samples for IL-1β and IL-12, and for 87 paired samples for IL-2 and IL-6 expression.

**Figure 1:**
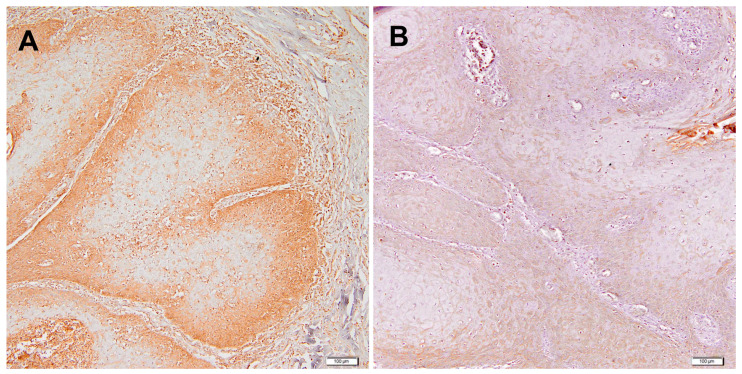
Representative examples of IL-12 immunohistochemistry staining: (**A**) Strong cytoplasmic positive reaction in tumour nests with additional staining in tumour-associated mononuclear inflammatory cells in the surrounding stroma. (**B**) Weak positive reaction in tumour cells with only sparse staining in stromal/inflammatory cells. Scale bar = 100 µm; original magnification ×200.

**Figure 2:**
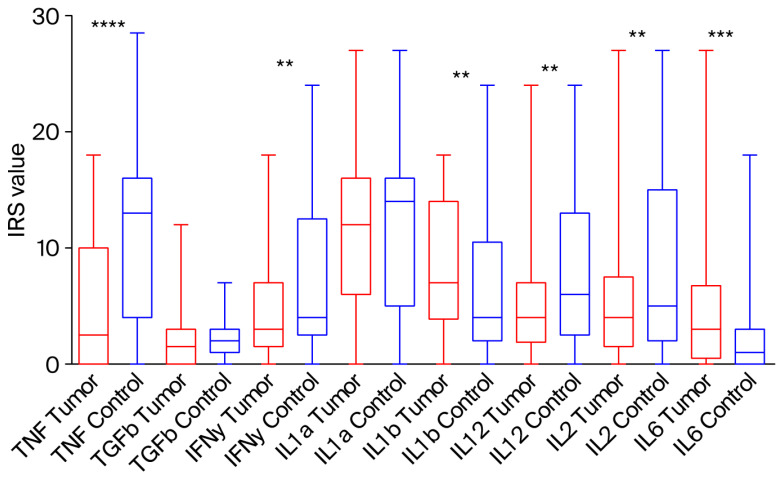
Comparison of cytokine expression between tumour tissue and adjacent healthy tissue from surgical margins in patients with PeCa. Box plots represent modified IRS of selected cytokines in tumour tissue (red) versus matched control tissue from the surgical margin (blue). Box represents 25–75% quartiles, while whiskers represent 5–95% percentile values. Statistically significant differences (** *p* < 0.01, *** *p* < 0.001, **** *p* < 0.0001; Wilcoxon signed-rank test) are indicated above the plot.

A representative IHC image illustrating the expression pattern observed in this study is shown for IL-12 (Figure 1). In tumour sections, IL-12 immunoreactivity was observed in tumour epithelial nests and in tumour-associated mononuclear inflammatory cells within the surrounding stroma and at the invasive front, whereas staining in the epithelium and stroma of negative surgical margins was generally weaker or absent. When the semi-quantitative IRS values were compared between tumour tissues and the corresponding margins, TNF-α, IFN-γ, IL-12 and IL-2 showed relatively lower expression in tumours, whereas IL-1β and IL-6 were elevated in tumour tissue (Figure 2). Since only limited data exist on the semi-quantitative IHC evaluation of these cytokines in PeCa, we next performed Receiver Operating Characteristic (ROC) curve analysis with the minimum distance method (i.e., the shortest Euclidean distance to the ideal point [0, 1]) to determine cut-off values for each cytokine relative to paired normal tissues (Table 2, Figure S3). Based on this approach, TNF-α, IL-6 and IL-12 showed the highest discriminative ability between tumour and normal tissue (AUC 0.76, 0.65 and 0.65, respectively; Table 2), while TGF-β1 and IL-1α yielded AUC values close to 0.5, indicating limited diagnostic value. These ROC-derived thresholds were subsequently used to dichotomise cytokine expression (high vs. low) in tumour samples for the association and survival analyses [14].

The IHC results for the tumour tissue divided into “low” and “high” immuno-expression are summarized in Table 3. Among the analysed cytokines, IL-1β (68.9%) and IL-6 (67.8%) showed the highest proportions of elevated expression, suggesting their prominent role in the tumour microenvironment. In contrast, IL-2 exhibited the lowest frequency of high expression (28.7%), indicating relatively limited activity in tumour tissue. The remaining cytokines displayed intermediate distributions, without clear predominance of high or low expression.

Several statistically significant associations were observed between cytokine expression levels and clinical parameters. IL-1α expression was markedly higher in tumours from patients aged ≥64 years (*p* = 0.021), and in advanced TNM prognostic stages (*p* = 0.047). Similarly, IL-12 expression was significantly elevated in invasive SCC compared to PeIN (*p* = 0.034), in advanced TNM prognostic stages (*p* = 0.0453), and in patients who died from penile cancer (*p* = 0.010). TGF-β1 expression was also significantly higher in advanced TNM prognostic stages (*p* = 0.028).

### 2.3. Cytokine Expression and TNM Stage and Grade

**Figure 3:**
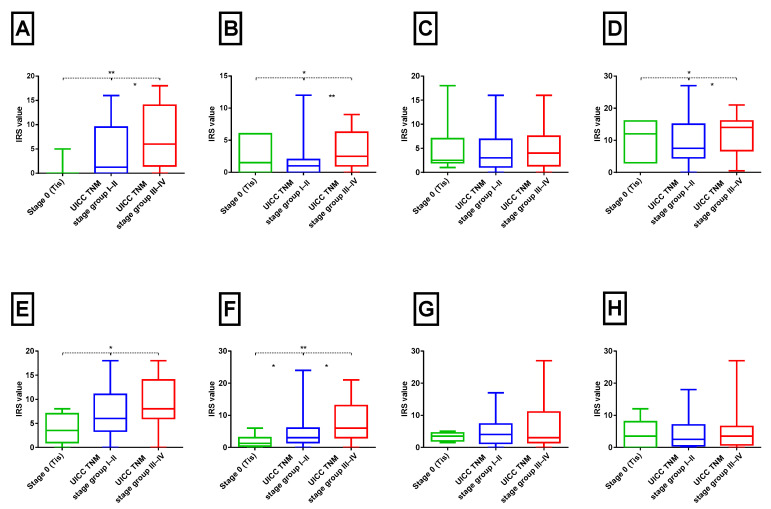
Immunohistochemistry expression of pro-inflammatory cytokines in PeCa tissues stratified by UICC TNM Stage group (stage 0 vs. I–II vs. III–IV). Box plots show modified IRS for TNF-α (**A**), TGF-β1 (**B**), IFN-γ (**C**), IL-1α (**D**), IL-1β (**E**), IL-12 (**F**), IL-2 (**G**), and IL-6 (**H**) across carcinoma in situ (Stage 0/Tis), early-stage (Stage I–II), and advanced-stage (Stage III–IV) tumours. Statistically significant differences between tumour stages are indicated above the plots (* *p* < 0.05, ** *p* < 0.01; Kruskal–Wallis with Dunn’s post hoc test). Boxes represent interquartile ranges (25–75%), and whiskers represent 5–95% values.

**Figure 4:**
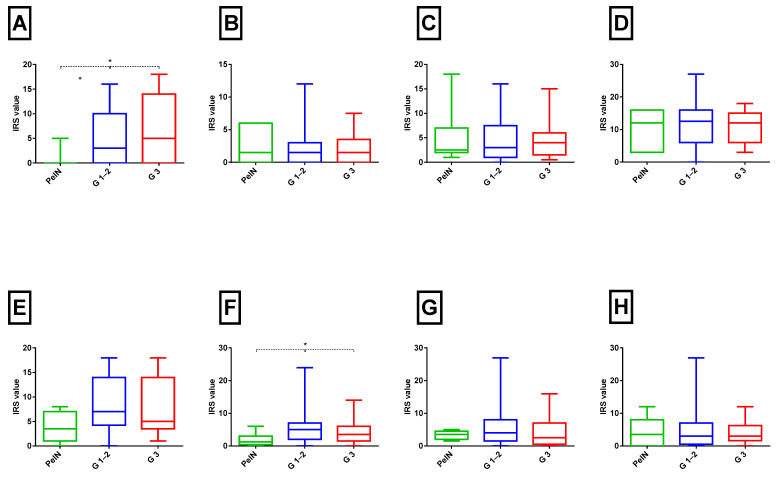
Immunohistochemistry expression of pro-inflammatory cytokines across tumour grade categories in PeCa samples. Box plots show modified IRS for TNF-α (**A**), TGF-β1 (**B**), IFN-γ (**C**), IL-1α (**D**), IL-1β (**E**), IL-12 (**F**), IL-2 (**G**), and IL-6 (**H**). Tissue samples were grouped as PeIN, low–intermediate grade (G1–G2), and poorly differentiated (G3) tumours. Boxes represent interquartile ranges (25–75%), whiskers represent the 5–95% values. Statistically significant differences between groups were assessed with the Kruskal–Wallis test followed by Dunn’s post hoc test (* *p* < 0.05).

The expression of cytokines in tumour samples in relation to the UICC TNM Stage/Prognostic Group (8th edition) and histological tumour grade (G) are shown in Figure 3 and Figure 4. Significant differences in expression were observed for TNF-α, TGF-β, IL-1α, IL-1β, and IL-12 between PeIN and early (I + II) and advanced-stage (III + IV) tumours (Kruskal–Wallis test, *p* < 0.05). For instance, TNF-α levels exhibited a progressive increase from Tis through early to advanced-stage tumours (Spearman’s correlation, ρ = 0.32, *p* < 0.05). Similar upward trends in advanced disease were noted for TGF-β, IL-1α, IL-1β, and IL-12, with weak but statistically significant positive correlations (Spearman’s correlation, r^2^ = 0.26, 0.23, 0.25, and 0.33, respectively; all *p* < 0.05).

In contrast, when analysed according to histological tumour grade, only TNF-α demonstrated a significant increase in expression in G1–2 and G3 tumours compared to PeIN (Mann–Whitney test, *p* < 0.05; Figure 4A). No statistically significant differences in the immuno-expression of other cytokines were detected. However, weak positive correlations were observed for the same proteins as in the UICC TNM Stage/Prognostic Groups comparison.

### 2.4. Cytokine Expression and Patient Survival

**Figure 5:**
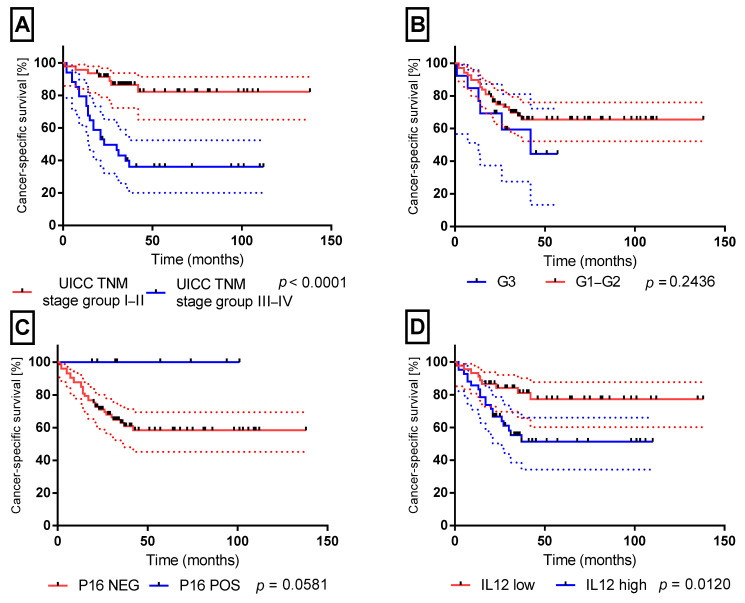
Kaplan–Meier cancer-specific survival (CSS) curves for patients with penile cancer (30 events). Analyses were performed according to (**A**) UICC TNM stage group, (**B**) histological grade, (**C**) p16 expression, and (**D**) IL-12 immuno-expression in tumour samples. Cut-off values for immunoreactivity were determined using the minimum distance method on ROC curves. Dashed lines indicate 95% confidence intervals. *p*-values were calculated using the log-rank (Mantel–Cox) test and are shown for each plot. According to the Union for International Cancer Control (UICC) TNM Classification of Malignant Tumours, 8th edition.

Because most patients in this cohort presented with early-stage disease and long-term follow-up revealed 5 of 35 deaths (14.3%) were unrelated to penile cancer, we analysed CSS rather than overall survival (OS) to better reflect the biological course of the disease. CSS was defined as the time from surgery to death from penile cancer, while deaths from other causes were censored at the time of occurrence. Survival analysis using the Mantel–Cox test revealed significantly reduced CSS in patients diagnosed with advanced-stage disease (Stage III + IV; *p* < 0.00001; Figure 5A). No significant correlation was identified with histological tumour grade (G; Figure 5B). A trend indicating improved survival in HPV-positive patients was noted (100% survival; Figure 5C), although this was not statistically significant (*p* = 0.0581). Importantly, increased IL-12 expression correlated with poorer CSS (Figure 5D). No other cytokines were significantly associated with survival.

### 2.5. Cox Hazard Model Analysis

Both univariable and multivariable Cox regression analyses identified TNM prognostic stage (Stage III–IV vs. Stage I–II) and elevated IL-12 expression in cancer tissues as independent predictors of a poor prognosis. In the multivariable model, IL-12 expression was associated with an approximately twofold increased risk of cancer-related mortality (HR 2.42, 95% CI: 1.08–5.41, *p* = 0.031). TNM prognostic stage was also a significant independent prognostic factor (HR 5.03, 95% CI: 2.12–11.95, *p* = 0.0002), as shown in Table 4. The analysis was based on 30 cancer-specific deaths, resulting in an EPV of 15 for the two-variable model, which exceeds the commonly recommended threshold of 10. To avoid overfitting, we did not include additional variables such as age, histological grade, or other ROC-significant cytokines (TNF-α, IL-6, IL-2; Table 2) in the primary multivariable model, as this would have reduced the EPV below 10. Instead, we tested these factors in exploratory multivariable Cox models, in which each covariate (age, histological grade, TNF-α, IL-6, IL-2) was added one at a time to the core model including TNM prognostic stage and IL-12 IRS cut-off. In none of these models did the additional covariate reach statistical significance, and the hazard ratios for TNM prognostic stage and IL-12 remained materially unchanged compared with the multivariable analysis presented in Table 4. Furthermore, bootstrap validation (1000 iterations) confirmed the stability of estimates, yielding HRs of 5.01 (95% CI: 2.10–11.90) for TNM prognostic stage and 2.40 (95% CI: 1.06–5.38) for IL-12 IRS cutoff, closely aligning with the original results. These findings support the robustness of the model despite the limited number of events, although the prognostic value of IL-12 should be regarded as exploratory and requires validation in larger, multicenter cohorts. Additionally, in exploratory univariable Cox models, cytokine immuno-expression in negative surgical margins was not significantly associated with CSS (Table S1), supporting our focus on tumour-based biomarkers.

## 3. Discussion

Our study investigated the expression of the pro-inflammatory cytokines IL-1A, IL-1B, IL-2, IL-6, IL-12, TGF-β1, TNF-α, and IFN-γ in penile SCC and their associations with disease progression and survival. Compared with paired surgical margins, tumour tissues exhibited an inflammatory shift characterised by increased IL-1β and IL-6 alongside reduced TNF-α, IFN-γ, IL-12 and IL-2. Importantly, IL-12 immuno-expression within tumour tissues—not in the surrounding margins—increased with advancing UICC TNM Stage/Prognostic Group and was independently associated with poorer CSS. IL-1α, IL-12, and TGF-β1 expression correlated significantly with age and TNM prognostic stage. In the present study, CSS was analysed instead OS to minimise confounding by non-cancer-related deaths and better reflect the biological aggressiveness of the disease. CSS analysis identified advanced TNM prognostic stage (stage III–IV) and increased IL-12 expression as independent predictors of poor prognosis, highlighting the potential role of pro-inflammatory cytokines in PeCa progression and their prognostic significance. Furthermore, in exploratory survival analyses, cytokine expression in negative surgical margins did not show clinically relevant associations with CSS (Table S1), which supports our emphasis on tumour-based biomarkers. Additionally, from diagnostic perspective, our ROC curve analysis indicated that TNF-α, IL-6 and IL-12 had the strongest ability to discriminate tumour tissue from the corresponding surgical margins (AUC 0.76, 0.65 and 0.65, respectively).

Several studies have suggested that immune and inflammatory pathways play critical roles in PeCa; however, comprehensive data remain limited. Transcriptomic analyses have demonstrated the elevated mRNA expression of IL-1A, IL-1B, IL-6, IFN-γ, and TGF-β1 in penile tumour tissue, with a positive correlation between IFN-γ levels and tumour stage [11]. Furthermore, Zhou et al. demonstrated that the elevated expression of IFN-γ in PeCa enhances the immunosuppressive function of indoleamine 2,3-dioxygenase (IDO1), which inhibits effector T-cell proliferation and facilitates regulatory T-cell differentiation. This process ultimately cultivates an immunosuppressive tumour microenvironment, thereby contributing to disease progression [15]. Additionally, the comprehensive genomic characterisation of PeCa cell lines identified frequent mutations and copy number alterations in key regulators of the TGF-β and Janus kinase/signal transducer and activator of transcription pathways. Specifically, recurrent alterations in transforming growth factor beta receptor type 2 (*TGFBR2*), SMAD family member 2 (*SMAD2*), and SMAD family member 4 (*SMAD4*) genes were detected, potentially impairing TGF-β signalling and underscoring the role of immune dysregulation in tumour biology [16]. Our study confirmed a marked upregulation of IL-1α and TGF-β1 in advanced and node-positive tumours. Conversely, IFN-γ, which has been previously linked to tumour progression at the mRNA level, exhibited diminished protein expression in tumours and did not show prognostic significance in our cohort. This finding suggests potential post-transcriptional or microenvironmental modulation of these genes. Furthermore, a methodological discrepancy between mRNA-based and protein-level analyses is evident, as our preliminary study showed [11] elevated mRNA levels without corresponding prognostic significance, highlighting the importance of tissue-level protein assessment in clinically relevant cohorts. Notably, we identified IL-12 as a marker associated with tumour invasiveness, lymph node metastasis, and cancer-specific survival. IL-12 is therefore shown to be an independent predictor of poor prognosis, indicating its potential utility both as a biomarker and a potential therapeutic target.

Interleukin 12 is a crucial pro-inflammatory cytokine that serves as a link between innate and adaptive immunity [17,18,19]. Structurally, IL-12 is a heterodimeric glycoprotein consisting of two covalently linked subunits, p35 and p40, both of which are essential for its biological function [17]. This cytokine primarily exerts its effects by inducing IFN-γ production and facilitating the proliferation and activation of NK cells and CD8+ cytotoxic T lymphocytes. IL-12 is predominantly secreted by dendritic cells, macrophages, monocytes, and B cells in response to microbial stimuli. Through the activation of its cognate receptor, composed of IL-12Rβ1 and IL-12Rβ2 subunits, IL-12 signals mainly via STAT4—a transcription factor necessary for its immunostimulatory functions [17]. IL-12 plays a pivotal role in directing the differentiation of naïve CD4+ T cells towards a Th1 phenotype while concurrently inhibiting Th2 polarisation. Under normal physiological conditions, IL-12 prompts T and NK cells to release IFN-γ, which subsequently triggers the expression of HLA-DR (MHC class II) on antigen-presenting cells. This mutual positive feedback loop between IL-12 and IFN-γ is crucial for efficient antigen presentation and T-cell activation, thereby facilitating strong immune recognition of tumour cells [18]. In the existing literature, there is a notable paucity of data concerning the expression of IL-12 in PeCa. Nevertheless, in various malignancies, such as glioblastoma, colorectal cancer, gastric carcinoma, head and neck squamous cell carcinoma, and renal cell carcinoma, IL-12 expression is frequently downregulated, which could facilitate tumour immune evasion. Preclinical models have demonstrated that the local delivery of IL-12 using adenoviral vectors, nanoparticles, or microspheres results in tumour regression, increased survival, and enhanced immune cell infiltration into the tumour microenvironment. Furthermore, the combination of IL-12 treatment with conventional therapies, including radiotherapy, chemotherapy, and immune checkpoint blockade, has exhibited synergistic effects, positioning IL-12 as a promising candidate for combination immunotherapy strategies [19]. Taken together, these experimental data and clinical observations have led to IL-12 being classically regarded as an anti-tumour cytokine, and reduced IL-12 expression or signalling is often associated with immune escape and poor prognosis in several solid malignancies.

Interestingly, our findings diverge from this classical view of IL-12 as predominantly anti-tumour. Whereas many studies in glioblastoma, colorectal, gastric and head and neck cancers report downregulation of IL-12 or associate higher IL-12 levels with improved tumour control and more favourable prognosis [17,18,19], we observed that in penile SCC tumour IL-12 immuno-expression was significantly higher in invasive and advanced TNM stages and independently predicted poorer CSS. From a morphological standpoint, IL-12 immunoreactivity in our cohort was localised to both tumour nests and tumour-infiltrating mononuclear inflammatory cells within the surrounding stroma and at the invasive front, suggesting that both neoplastic and immune components may contribute to the IL-12 signal. However, because we used single-marker chromogenic IHC without double staining or compartment-specific scoring, these observations only indicate the spatial distribution of IL-12 protein and do not allow us to determine which cell types are the predominant source of IL-12 production or secretion. We therefore interpret elevated tumour IL-12 in PeCa as a context-dependent, largely non-protective signal that likely reflects late, ineffective Th1-type activation and chronic tumour-associated inflammation rather than a sustained, successful anti-tumour response. In advanced and ulcerated penile tumours, persistent epithelial injury, HPV infection and the continuous recruitment and stimulation of myeloid cells, together with secondary bacterial colonisation, may all provide strong stimuli for IL-12 induction. In this setting, IL-12 may function less as a direct effector cytokine mediating tumour clearance and more as a surrogate marker of dysregulated immunity and a hostile, inflamed microenvironment that favours tumour progression. This model remains speculative and underscores the need for future studies combining multiplex immunohistochemistry, detailed immune phenotyping and microbiological assessment to dissect the cellular sources and functional roles of IL-12 in PeCa. Interleukin-1α and IL-1β are key members of the IL-1 family that orchestrate inflammatory and fibrotic responses and are produced by macrophages, keratinocytes and endothelial cells. Through IL-1R1 signalling, they induce downstream transcriptional programmes that regulate inflammation, cell proliferation and survival, and can thereby contribute to tumourigenesis [20,21,22]. In the context of cancer, IL-1 signalling promotes tumour progression by upregulating matrix metalloproteinases, stimulating pro-angiogenic mediators such as VEGF and prostaglandin E2, and enhancing adhesion molecule expression on endothelial cells, which facilitates tumour cell extravasation and metastasis. IL-1α and IL-1β also recruit and polarise tumour-associated macrophages and myeloid-derived suppressor cells, reinforcing an immunosuppressive microenvironment [20,23]. The roles of IL-1A and IL-1B have been extensively studied in various cancers, including non-small-cell lung cancer (NSCLC), head and neck squamous cell carcinoma, melanoma, colorectal adenocarcinoma, glioma, and cervical cancer [24,25,26,27]. In advanced NSCLC, high IL-1β levels were identified as an adverse prognostic factor associated with shorter progression-free and OS [24], while in head and neck cancer elevated IL-1α correlated with an increased risk of distant metastasis [25]. Metastatic NSCLC, colorectal cancer and melanoma samples also show increased IL-1 transcript levels [22], and in glioma stem-like cells prolonged IL-1β exposure promotes tumour progression via COX-2–mediated oxidative DNA damage and disruption of p53 tumour-suppressor activity [26]. In contrast, reduced IL-1β mRNA expression in cervical tissue has been linked to the progression of HPV-related precancerous lesions, suggesting a context-dependent, potentially protective role of IL-1β in the early stages of carcinogenesis [27].

Nonetheless, the available data regarding the significance of IL-1A and IL-1B in PeCa remain limited [10]. Our findings confirm that penile cancer tissues with elevated IL-1α expression are significantly correlated with advanced TNM prognostic stages. Consequently, IL-1A may serve as a potential negative prognostic marker of PeCa. These results suggest that in PeCa, IL-1α predominantly functions as an inducer of tumour-promoting inflammation rather than a protective pro-inflammatory signal. This perspective likely arises from a chronic inflammatory state driven by persistent HPV infection, epithelial cell damage, and the continuous recruitment of myeloid cells. These factors are major contributors to immune system dysfunction, disease progression, and treatment resistance. Consequently, IL-1α may serve as an indicator of immune system failure and a potential target for immunomodulatory therapy in patients with penile cancer.

TGF-β is a multifunctional cytokine with context-dependent roles in cancer. In early carcinogenesis it can act as a tumour suppressor by inhibiting epithelial proliferation and inducing apoptosis, but in advanced disease it typically promotes progression by driving epithelial–mesenchymal transition, angiogenesis and extracellular matrix remodelling, while at the same time exerting potent immunosuppressive effects. Through SMAD-dependent and SMAD-independent pathways, TGF-β dampens cytotoxic T-cell and NK-cell activity, reduces the production of key immunostimulatory cytokines such as IL-12 and IFN-γ, and favours the accumulation of regulatory T cells and tumour-associated macrophages, thereby creating a tolerogenic tumour microenvironment [28,29]. In various solid tumours, TGF-β1 overexpression has been consistently linked to tumour progression and adverse outcomes. In pancreatic cancer, all three TGF-β isoforms are upregulated at the protein and mRNA level and correlate with reduced survival [30]. In renal cell carcinoma, metastatic disease is characterised by significantly higher plasma concentrations of both latent and active TGF-β1 than localised tumours, and TGF-β1 drives adhesion and extracellular matrix-remodelling gene programmes associated with poor prognosis [31,32]. Additional layers of regulation include miR-34b, which modulates TGF-β signalling components and suppresses proliferation, migration and invasion in prostate cancer cells when upregulated [33], and the Int7G24A intronic variant of TGFBR1, which has been associated with increased susceptibility to bladder transitional cell carcinoma and RCC [34]. However, the role of TGF-β in PeCa has not been comprehensively characterised, except for two studies conducted by Czajkowski et al. [11] and Zhou et al. [16]. In our cohort, TGF-β1 was overexpressed in 53.76% of the cases of penile cancer and its elevated expression significantly correlated with lymph node metastasis (*p* = 0.028). This finding supports its role in facilitating the invasive and metastatic behaviour of PeCa. These results suggest that TGF-β may be crucial in creating an immunosuppressive environment within PeCa. The consistent elevation of its levels in the advanced stages of the disease indicates its potential as a prognostic biomarker and therapeutic target for PeCa.

In our study, IL-2, IL-6, TNF-α, and IFN-γ levels were not significantly associated with clinicopathological characteristics or CSS. IL-2 exhibited high IHC expression (28.74%) in tumour samples without statistically significant associations were found between IL-2 expression and clinicopathological parameters. These findings may depend on the complex and dualistic role of IL-2, which stimulates effector T cells while also promoting immune tolerance by activating regulatory T cells [35]. Similarly, the contradictory effects observed may be attributed to TNF-α, whose IHC expression was detected in only 39.36% of cases and had no correlation with PeCa prognosis in our study. This variability may be due to the dual role of TNF-α, which can exhibit either pro-apoptotic or tumour-promoting effects depending on the local immune and molecular environment [36]. Despite IHC analysis showing a high expression level (67.82%) of interleukin-6 (IL-6) in PeCa samples, no significant correlations were observed between IL-6 levels and clinicopathological variables. Rather than suggesting a tumour-specific effect, this observation may underscore the pleiotropic and systemic functions of IL-6 in inflammation. In contrast to cancers such as colorectal or pancreatic cancer, where IL-6 facilitates epithelial–mesenchymal transition and metastasis, its presence in penile cancer might indicate a reactive inflammatory response that lacks prognostic significance [10]. Finally, IFN-γ with 54.84% of IHC expression in PeCa without any prognostic value in our study, may depend on the context-dependent role of IFN-γ. Generally, IFN-γ inhibits tumour growth; however, dysregulated IFN-γ signalling may paradoxically contribute to immune escape by promoting tumour cell immunoediting and reducing immunogenicity. Therefore, the expression of IFN-γ may not be the direct cause of PeCa aggressiveness or prognosis but rather represents a dynamic balance between immune activation and tolerance [37]. Together, these findings emphasise the complexity and context of pro-inflammatory cytokine signalling in PeCa biology.

In interpreting our findings, it is important to consider the characteristics of the studied cohort. In addition to classical prognostic factors such as stage, nodal status, and histological grade, several molecular and viral markers which could vary across geographical regions have been recognised as relevant in PeCa [1,2,4,10]. In our cohort, p16 positivity, a surrogate marker for transcriptionally active high-risk HPV, was observed in 10.6% of patients, which is in line with other reports from Europe [4]. However, higher rates have been reported in endemic regions such as South America or India, where HPV positivity may exceed 30–50% [1]. The prognostic relevance of HPV/p16 status is increasingly recognised, as highlighted by the recent ISUP consensus [38]. Additionally, meta-analytic data indicate that positive p16 status and HPV DNA positivity are associated with improved cancer-specific survival in penile cancer, with p16 positivity also correlating with better overall survival, underscoring its prognostic value [39]. In contrast, HPV positivity does not invariably confer a favourable prognosis. Recent genomic data demonstrate that TP53 loss-of-function mutations are associated with an aggressive, immune-excluded phenotype and poor outcomes, irrespective of HPV status [40]. In our cohort, HPV-positive status (10.6% of cases) was associated with a trend towards improved cancer-specific survival (100% CSS), but this did not reach statistical significance due to the small sample size. Thus, the prognostic role of HPV in penile cancer in a Central European population remains inconclusive. Beyond viral and TP53-related alterations, emerging genomic changes such as Loss of Y chromosome (LOY) have recently been implicated in urological cancers, including prostate and bladder cancer, where they are associated with immune dysregulation and adverse outcomes. Although LOY has not yet been systematically studied in penile cancer, its potential role highlights the need to integrate viral, molecular, and immunological factors into future prognostic models [41]. Collectively, these insights support the concept that HPV status, TP53 alterations, and LOY, together with immunological markers such as IL-12, interact to shape the tumour microenvironment and may ultimately influence immune responsiveness and clinical outcomes.

This study is the first to identify associations between the expression of IL-1α, IL-12, and TGF-β1 and clinicopathological parameters in PeCa. While univariable analyses involved multiple comparisons across 11 parameters, increasing the risk of type I errors, the robustness of the findings was supported by multivariable Cox regression and bootstrap validation (1000 iterations), which consistently confirmed TNM prognostic stage and IL-12 IRS cutoff as significant predictors. Given the retrospective single-centre design and the limited number of cancer-specific events, this adverse prognostic association of tumour IL-12 should be regarded as exploratory and hypothesis-generating. Survival analysis further indicated that advanced TNM prognostic stage and elevated IL-12 expression were independent indicators of poor prognosis. Thus, this study provides evidence of immuno-dysregulation in penile cancer. Moreover, IL-1α, IL-12, and TGF-β1 may function as biomarkers and potential therapeutic targets. Despite the strengths and novelty of this study, certain limitations must be acknowledged. The retrospective, single-centre design and the restriction of our cohort to White Caucasian men treated at a single tertiary centre in Central Europe may limit the generalisability of these findings. In addition, the reliance on IHC analysis restricts the assessment of the dynamic or functional aspects of cytokine signaling in penile cancer. The absence of data of complementary mRNA expression, immune cell phenotyping, checkpoint molecule expression (e.g., PD-1/PD-L1), and stromal composition limits our interpretation. Additionally, the small number of HPV-positive cases prevented significant subgroup analyses to be conducted based on molecular aetiology. Furthermore, our study did not address other molecular alterations such as TP53 mutations or Loss of Y chromosome, which—together with HPV status and immunological markers like IL-12—may interactively shape the tumour microenvironment and ultimately influence immune responsiveness and clinical outcomes. Moreover, our study is lack of microbiological assessment which could have helped to further clarify the potential role of secondary bacterial infections in IL-12 upregulation. Another limitation is that our semi-quantitative IRS captured global cytokine expression within the tumour area and did not discriminate between tumour cells and immune or stromal compartments; as a result, we cannot determine whether the prognostic impact of IL-12 is driven predominantly by tumour cells, infiltrating myeloid cells, or other cell types. Finally, although CSS was used to reduce the confounding effect of non-cancer-related deaths, this approach may still be limited by the retrospective determination of the cause of death and the lack of progression-free survival data. Future studies should integrate tissue-based analyses with transcriptomic data and serum cytokine measurements to better understand the immune landscape of penile cancer. Moreover, functional studies are necessary to investigate whether inhibiting IL-1α, IL-12, or TGF-β1 can reduce tumour cell growth, migration, or invasion in vitro, and to examine their role in tumour progression in animal models. Further research using multiplex immunofluorescence or flow cytometry could help identify key immune cells in the tumour microenvironment and their relationship with cytokine expression and patient outcomes. Future studies with larger cohorts will be essential to confirm these results and to apply formal multiple comparison adjustments, such as Bonferroni or false discovery rate (FDR) corrections, while refining cut-off selection with both statistical and clinical considerations.

## 4. Materials and Methods

### 4.1. Patients and Samples

This retrospective study included paired samples of PeCa tumour tissues and corresponding negative surgical margins, collected from 94 male patients of White Caucasian who had undergone surgical treatment (glansectomy, partial or total penectomy) at the tertiary Department of Urology between January 2014 and September 2024. Histopathological types other than the usual SCC were excluded. The study was approved by the university’s Ethics Committee (decision no. NKBBN/168/2023), and written informed consent was obtained from all patients before surgery. The histological subtypes of PeCa were classified according to the 2022 World Health Organization (WHO) Classification [13]. PeIN lesions were diagnosed and graded according to the 2022 WHO Classification of Tumours of the Urinary System and Male Genital Organs [13]; in this cohort, only high-grade lesions (PeIN grade III) were present. Pathological staging was performed using the 8th edition of the TNM Classification of Malignant Tumours [42].

### 4.2. TNM Stage Grouping

Tumour staging was performed according to the 8th edition of the UICC TNM Classification of Malignant Tumours [42]. For analytical purposes, patients were stratified into two prognostic groups reflecting disease advancement: Stage I–II (T1–T3N0M0), representing localised disease, and Stage III–IV (T1–4N1–3M0 or any M1), representing regionally advanced or metastatic disease. This grouping aligns with the TNM-based prognostic categories and was used in the Cox regression model to ensure adequate event numbers per variable. Seven patients diagnosed with carcinoma in situ (Tis/PeIN) were excluded from survival analyses due to the absence of invasive disease or cancer-specific events. Among the ten patients with pNx status, seven also had Tis lesions. All pNx cases were clinically node-negative (cN0) and were therefore included in descriptive statistics but excluded from survival analyses to avoid potential bias related to unverified nodal status.

### 4.3. Immunohistochemistry

IHC staining was performed as previously described by Kiezun et al. [43], with modifications. The sections (4 μm thick) were subjected to an antigen retrieval procedure by microwaving for 4 min in Retrieval Solution Buffer at pH 6.0 for IL1-α, IL1-β, IL-2, IL-6, and IL-12 and pH 9.0 for TGF-β1, IFN-γ, and TNF-α (Leica Microsystems, Wetzlar, Germany). The samples were then incubated with 3% H_2_O_2_ in methanol for 10 min, and then the unspecific binding sites were blocked with 2.5% normal horse serum (Vector Laboratories, Burlingame, CA, USA) for 30 min. All primary antibodies were purchased from Affinity Bioscience (Cincinnati, OH, USA). The sections were incubated overnight at 4 °C with rabbit polyclonal anti-human antibodies against IL1-α, IL1-β, IL-2, IL-6, IL-12, TGF-β1, IFN-γ, and TNF-α (diluted in PBS: 1:400, DF6893; 1:150, AF5103; 1:400, DF6095; 1:200, DF6087; 1:400, AF5133; 1:400, AF1027; 1:400, DF6045; 1:400, AF7014, respectively). The next day, the sections were incubated for 30 min with commercially diluted secondary antibodies (ImmPRESS Universal reagent Anti-Mouse/Rabbit Ig; Vector Laboratories, Burlingame, CA, USA). To visualise the immunoreaction, the sections were immersed in DAB (Agilent Technologies, Santa Clara, CA, USA) and then counterstained with haematoxylin (Merck, Rahway, NJ, USA). The sections were dehydrated in ethanol, cleared in xylene, and mounted. The specificity of IHC staining was checked by omitting the primary antibody and replacing it with the same dilution of rabbit serum. The immuno-stained sections were photographed using an Olympus BX-53 microscope with an XC-50 camera (Olympus Corp., Tokyo, Japan). Representative examples of IHC stainings for all investigated molecules are provided as Figures S1 and S2 in the Supplementary Material.

### 4.4. Immunohistochemistry Reactivity Scoring Method

The immunoreactivity of the examined cytokines was evaluated by two independent pathologists who were blinded to the patients’ clinical data using the modified Remmele Immunoreactive Score (IRS) based on the number of cells showing immunoreactivity (0, no reaction; 1, ≤10%; 3, 11–30%; 6, 31–60%; 8, 61–80%; and 10, >80%) and the reaction intensity (0, no reaction; 1, weak; 2, mild; 3, strong), as described previously [44]. Thus, a score of 0–30 was obtained for each section cases with heterogeneous staining were evaluated according to the predominant staining intensity, representing more than 50% of the positive cells. Focal areas with stronger or weaker staining were not scored separately but contributed to the proportion score [45]. In case of discrepant assessments made by the two pathologists, a third independent scientist unaware of the patient characteristics and the two prior assessments also evaluated the sections. For all cytokines, immunoreactivity was assessed within the tumour region, encompassing staining in tumour cells as well as in tumour-associated stromal and inflammatory cells. Compartment-specific scoring (epithelium vs. immune/stromal) was not performed, as heterogeneous patterns were integrated into a single modified IRS value per case.

### 4.5. Statistical Analysis

Statistical analyses were performed using GraphPad Prism ver. 6.07 (GraphPad Software, San Diego, CA, USA), Statistica ver. 13.3 (TIBCO Software Inc., Palo Alto, CA, USA), and R software ver. 4.4.1 (R Core Team, R Foundation for Statistical Computing, Vienna, Austria) with the survival package. For paired samples, the Wilcoxon signed-rank test was applied, while unpaired comparisons were evaluated with the Mann–Whitney U test followed by Dunn’s post hoc test. Multiple-group comparisons were analyzed with the Kruskal–Wallis test, and correlations were assessed using Spearman’s rank test. To evaluate discriminative ability, ROC curve analysis with the minimum distance method was applied, defining cut-off values by minimizing the Euclidean distance between each point on the ROC curve and the ideal classifier (0, 1) [14,46]. CSS was analyzed with Kaplan–Meier estimates and the log-rank (Mantel–Cox) test. Cox proportional hazards regression was performed in a two-step approach (univariable, then multivariable), and internal validation was conducted using bootstrap resampling (1000 iterations). Results are presented as hazard ratios (HRs) with 95% confidence intervals (CIs). A *p*-value < 0.05 was considered statistically significant. In exploratory analyses, we additionally tested cytokine IRS values in negative surgical margins as predictors of CSS using univariable Cox models (Table S1).

## 5. Conclusions

The elevated immuno-expression of IL-1α, IL-12, and TGF-β1 in tumour tissues was significantly associated with advanced tumour stages and nodal involvement. Notably, increased tumour IL-12 immuno-expression emerged, in this exploratory analysis, as an independent indicator of poor cancer-specific survival, suggesting that IL-12 may be a promising prognostic biomarker and potential therapeutic target in PeCa; however, these findings require validation in larger, preferably multicentre cohorts. Conversely, the identification of the roles of IL-2, IL-6, IFN-γ, and TNF-α was limited in our study, highlighting the necessity for functional and mechanistic validation in future studies.

## Figures and Tables

**Table 1:** Clinicopathological characteristics of the patients with penile squamous cell carcinoma included in this study.

Patients	Criteria	Count	Percent [%]
**Gender**	Male	94	100%
**Race/ethnicity**	White Caucasian	94	100%
**Age (y.o.)**	Minimum	34	-
	Maximum	84	
	Median	64	
**BMI [kg/m^2^]**	Minimum	17	-
	Maximum	38	
	Median	29	
**TNM—Tumour**	Tis	7	7.45%
	pT1a	29	30.85%
	pT1b	13	13.83%
	pT2	32	34.04%
	pT3	12	12.77%
	pT4	1	1.06%
**TNM—Nodes**	pNx	10	10.63%
	pN0	50	53.2%
	pN1	10	10.63%
	pN2	12	12.77%
	pN3	12	12.77%
**TNM—Metastases**	M0	93	98.94%
	M1	1	1.06%
UICC TNM stage group *	Stage 0 (pTis)	7	7.45%
	Early I + II	34	36.2%
	Advanced III + IV	53	56.4%
**Grading**	PeIN	7	7.45%
	G1	21	22.34%
	G2	49	52.12%
	G3	17	18.09%
Histopathological type **	Squamous cell carcinoma (usual type)	94	100%
**p16 status**	Positive	10	10.64%
	Negative	84	89.36%
**Survival status at last follow-up**	Alive	59	62.8%
	Deceased	35	37.2%
**Follow-up** **(months)**	Minimum	3	
	Maximum	138	
	Median	36.5	

* According to the Union for International Cancer Control (UICC) TNM Classification of Malignant Tumours, 8th edition.** According to the 2022 WHO Classification of Tumours of the Urinary System and Male Genital Organs.

**Table 2:** Results of the ROC analyses between IRS results of paired tumour–normal tissues of PeCa patients.

ROC Analysis	TNF-α	TGF-β	IFN-γ	IL-1a	IL-1b	IL-12	IL-2	IL-6
**AUC**	0.7639	0.5117	0.5909	0.5098	0.6149	0.6513	0.5948	0.6523
**Std. Error**	0.03402	0.04285	0.04175	0.04233	0.04217	0.04045	0.04338	0.04177
**95%CI**	0.6972–0.8306	0.4277–0.5957	0.5090–0.6727	0.4268–0.5928	0.5322–0.6976	0.5720–0.7306	0.5097–0.6798	0.5704–0.7342
***p* value**	<0.0001	0.7822	0.0323	0.8166	0.0078	0.0004	0.0308	0.0005
**Cut-off value in tumour samples**	4.5	1.75	2.75	13.5	4.5	4.25	6.75	1.25

**Table 3:** Associations between high or low immunohistochemistry expression of selected cytokines in tumour samples and clinical–pathological parameters in patients with PeCa. The frequencies of high and low IHC expression are shown alongside the corresponding *p*-values for age, tumour type (PeIN vs. Ta-T4 SCC), TNM prognostic stage (I + II vs. III + IV), histological grade (G1–2 vs. G3), p16 status, and CSS. A 2 × 2 Fisher’s exact test was applied.

Cytokine	High (*n*/%)	Low (*n*/%)	Age < 64/≥64 (*p*-Value)	PeCa Type: PeIN vs. Ta-T4 SCC (*p*-Value)	UICC TNM Stage Group *: I + II vs. III + IV (*p*-Value)	Grade: G1–2 vs. G3 (*p*-Value)	p16 Status (*p*-Value)	Alive vs. Deceased (*p*-Value)
**TNF-α**	37 (39.36%)	57 (60.64%)	0.294	0.144	0.264	0.389	1.000	0.486
**TGF-β1**	50 (53.76%)	43 (46.24%)	0.537	1.00	**0.028**	1.00	1.000	0.253
**IFN-γ**	51 (54.84%)	42 (45.16%)	0.835	0.697	0.386	0.781	0.177	0.252
**IL-1a**	41 (43.62%)	53 (56.38%)	**0.021**	0.463	**0.047**	0.779	1.000	0.352
**IL-1b**	62 (68.89%)	28 (31.11%)	0.494	0.198	0.090	0.119	1.000	0.199
**IL-12**	39 (43.33%)	51 (56.67%)	0.143	**0.034**	**0.0453**	0.392	0.289	**0.010**
**IL-2**	25 (28.74%)	62 (71.26%)	0.813	0.315	0.807	0.765	1.0000	0.114
**IL-6**	59 (67.82%)	28 (32.18%)	0.065	0.655	0.339	0.531	0.427	0.803

* According to the Union for International Cancer Control (UICC) TNM Classification of Malignant Tumours, 8th edition.

**Table 4:** Univariate and multivariate Cox regression analyses for CSS in patients with penile cancer. Hazard ratios (HRs) with 95% confidence intervals (CIs) are presented for the TNM prognostic stage, histological, virological (p16) and IHC parameters. The significant predictors of survival are highlighted.

Parameter, 84 Patients, 30 Deaths Related to PeCa	Univariable Analysis			Multivariable Analysis		
	X^2^	*p*-Value	HR (95% CI)	X^2^	*p*-Value	HR (95% CI)
Age >64 vs. ≤64 [y]	1.26	0.26	0.64 (0.30–1.38)			
UICC TNM stage group * (Stage III–IV vs. Stage I–II)	**15.30**	**0.00009**	**5.54 (2.35–13.07)**	**13.43**	**0.0002**	**5.03 (2.12–11.95)**
Histological grade G3 vs. G1+2	1.31	0.25	1.69 (0.68–4.19)			
HPV infection NO vs. YES	1.21	0.27	3.07 (0.41–22.66)			
TNF-α IRS cutoff [4.5] ↓ vs. ↑	0.47	0.49	0.77 (0.36–1.62)			
TGF-β IRS cutoff [1.75] ↓ vs. ↑	0.96	0.32	0.68 (0.32–1.44)			
IFN-γ IRS cutoff [2.75] ↑ vs. ↓	1.38	0.23	0.62 (0.28–1.36)			
IL1-α IRS cutoff [13.5] ↑ vs. ↓	0.87	0.35	0.70 (0.33–1.47)			
IL1-β IRS cutoff [4.5] ↓ vs. ↑	1.54	0.21	0.54 (0.20–1.42)			
IL-12 IRS cutoff [4.25] ↑ vs. ↓	**6.49**	**0.01**	**2.83 (1.27–6.33)**	**4.63**	**0.031**	**2.42 (1.08–5.41)**
IL-2 IRS cutoff [6.75] ↑ vs. ↓	2.52	0.11	0.53 (0.25–1.55)			
IL-6 IRS cutoff [1.25] ↓ vs. ↑	0.25	0.61	0.80 (0.35–1.84)			

Cut-offs were determined by ROC analysis using the minimum distance method. Direction of comparisons (↑ vs. ↓) reflects the dichotomisation according to these thresholds. * According to the Union for International Cancer Control (UICC) TNM Classification of Malignant Tumours, 8th edition.

## Data Availability

The original contributions presented in this study are included in the article. Further inquiries can be directed to the corresponding author.

## Supplementary Materials

The following supporting information can be downloaded at: https://www.mdpi.com/article/10.3390/ijms262411829/s1.

## Abbreviations

BMI	Body mass index
CI	Confidence interval
CSS	Cancer-specific survival
HPV	Human papillomavirus
HR	Hazard ratio
IHC	Immunohistochemistry
IFN-γ	Interferon gamma
IL	Interleukin
IL-1α/IL-1A	Interleukin 1 alpha
IL-1β/IL-1B	Interleukin 1 beta
IL-2	Interleukin 2
IL-6	Interleukin 6
IL-12	Interleukin 12
IRS	Immunoreactive Score
MHC	Major histocompatibility complex
NK cell	Natural killer cell
OS	Overall survival
PeCa	Penile cancer
PeIN	Penile intraepithelial neoplasia
SCC	Squamous cell carcinoma
STAT	Signal transducer and activator of transcription
TAMs	Tumour-associated macrophages
TGF-β/TGF-β1	Transforming growth factor beta/beta 1
TGFBR	Transforming growth factor beta receptor
TNF-α	Tumour necrosis factor alpha
TNM	Tumour–Node–Metastasis classification
WHO	World Health Organization
