# Supplementary material for: IL-12 as a Potential Prognostic Marker in Penile Cancer: Implications for Immune Dysregulation
Source: Int J Mol Sci. 2025 Dec 7;26(24):11829. doi: 10.3390/ijms262411829 (PMC12732597; doi:10.3390/ijms262411829)
Supplement: Supplementary file 1 [file ijms-26-11829-s001.zip › ijms-3981017-supplementary.pdf]

Supplementary Materials

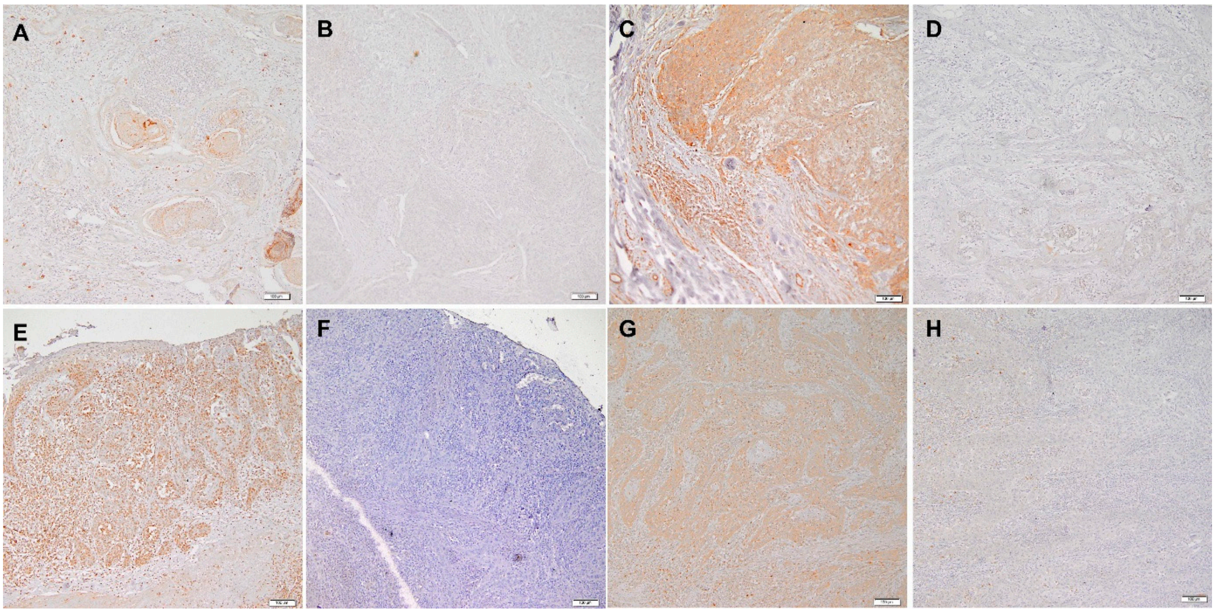

Figure S1: Representative examples of immunohistochemical stainings for IL6 (A weak reaction in tumour cells and scattered positive immune cells, B - negative reaction), IL2 (C - strong, positive reaction tumour cells and immune cells, D - negative reaction), IL1alpha (E - strong, positive reaction tumour cells and immune cells, F - negative reaction), and IL1beta (G - moderate, positive reaction tumour cells and some immune cells, H - negative reaction in tumour cells, weak positive reaction in immune cells). Scale bar = 100 μm; original magnification ×200.

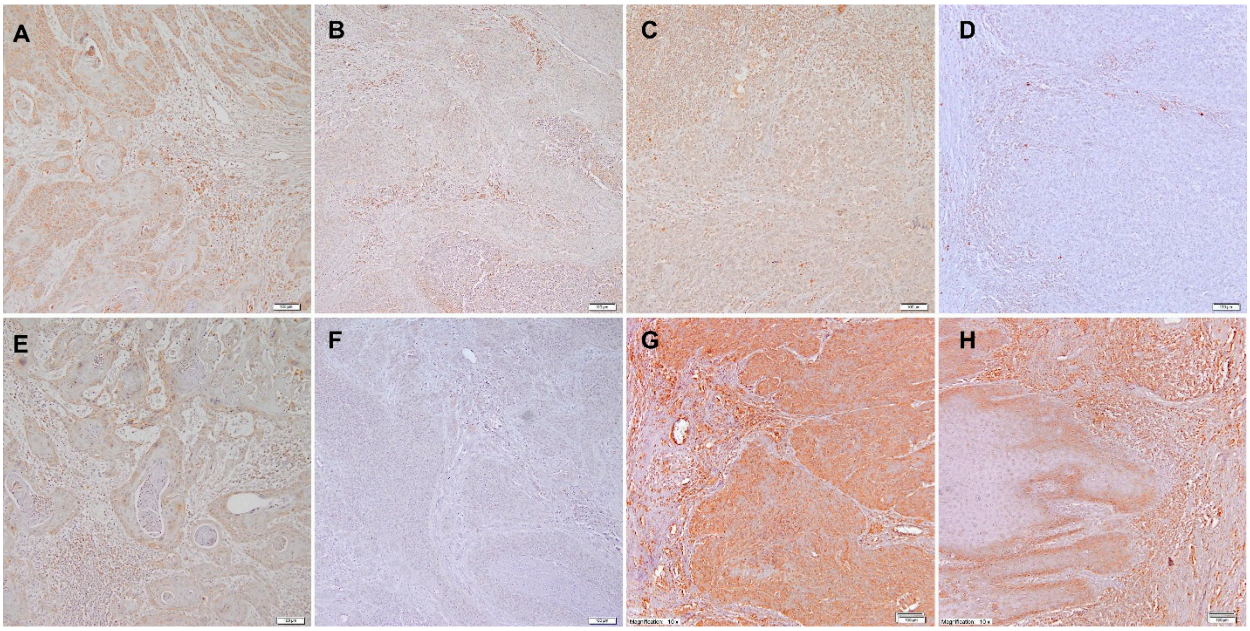

Figure S2: Representative examples of immunohistochemical stainings for IFN (A positive reaction in tumour cells and immune cells, B - negative reaction in tumour cells, weak positive reaction in immune cells), TNF (C – positive reaction tumour cells and immune cells, D - negative reaction in tumour cells, weak positive reaction in immune cells), TGF (E - weak positive reaction tumour cells and

immune cells, F - negative reaction), and IL12 (G – positive reaction tumour cells and immune cells, H - weak positive reaction in peripheral tumour cells and positive reaction in immune cells). Scale bar = 100 μm; original magnification ×200.

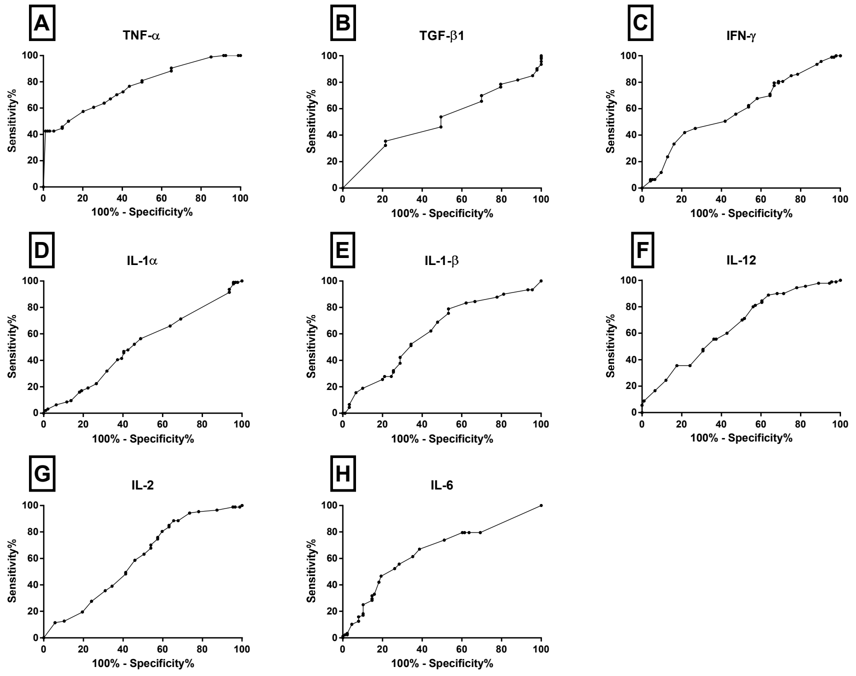

Figure S3: Receiver operating characteristic (ROC) curves for immunoreactive scores (IRS) of pro-inflammatory cytokines in penile cancer (PeCa) compared with corresponding negative surgical margins. ROC curves are shown for (A) TNF-α, (B) TGF-β1, (C) IFN-γ, (D) IL-1α, (E) IL-1β, (F) IL-12, (G) IL-2, and (H) IL-6. Optimal IRS cut-off values used to dichotomise tumour expression (high vs. low) in subsequent analyses were obtained with the minimum distance method (shortest Euclidean distance to the ideal classifier [0,1]).

Table S1. Univariable Cox regression analyses for cancer-specific survival (CSS) according to cytokine immunoexpression in negative surgical margins in patients with penile squamous cell carcinoma (PeCa). Hazard ratios (HRs) with 95% confidence intervals (CIs) are shown for high vs. low modified immunoreactive scores (IRS). Cut-off values were defined as described for tumour tissues in Table 2.

**Parameter, 84 patients, 30 deaths related to PeCa**	**Univariable Analysis**		
	**X²**	***p*-Value**	**HR (95% CI)**
TNF-α IRS cutoff [4.5] ↓ vs. ↑	0.191	0.662	0.818 (0.331 – 2.016)
TGF-β IRS cutoff [1.75] ↓ vs. ↑	1.332	0.248	0.640 (0.299 – 1.366)
IFN-γ IRS cutoff [2.75] ↑ vs. ↓	0.122	0.727	1.165 (0.494 – 2.747)
IL1-α IRS cutoff [13.5] ↑ vs. ↓	1.045	0.307	1.486 (0.695 – 3.76)
IL1-β IRS cutoff [4.5] ↓ vs. ↑	0.845	0.358	1.443 (0.661 – 3.151)

IL-12 IRS cutoff [4.25] ↑ vs. ↓	0.446	0.504	0.775 (0.366 – 1.639)
IL-2 IRS cutoff [6.75] ↑ vs. ↓	3.582	0.058	2.153 (0.973 – 4.764)
IL-6 IRS cutoff [1.25] ↓ vs. ↑	0.05	0.823	1.092 (0.504 – 2.367)
